# Fallers after stroke: a retrospective study to investigate the combination of postural sway measures and clinical information in faller’s identification

**DOI:** 10.3389/fneur.2023.1157453

**Published:** 2023-04-27

**Authors:** Johanna Jonsdottir, Fabiola Giovanna Mestanza Mattos, Alessandro Torchio, Chiara Corrini, Davide Cattaneo

**Affiliations:** ^1^IRCCS Fondazione Don Carlo Gnocchi, Milan, Italy; ^2^Department of Pathophysiology and Transplantation, University of Milan, Milan, Italy

**Keywords:** stroke, falls, balance, postural sway, stabilometric assessment

## Abstract

**Background:**

Falls can have devastating effects on quality of life. No clear relationships have been identified between clinical and stabilometric postural measures and falling in persons after stroke.

**Objective:**

This cross-sectional study investigates the value of including stabilometric measures of sway with clinical measures of balance in models for identification of faller chronic stroke survivors, and the relations between variables.

**Methods:**

Clinical and stabilometric data were collected from a convenience sample of 49 persons with stroke in hospital care. They were categorized as fallers (*N* = 21) or non-fallers (*N* = 28) based on the occurrence of falls in the previous 6 months. Logistic regression (model 1) was performed with clinical measures, including the Berg Balance scale (BBS), Barthel Index (BI), and Dynamic Gait Index (DGI). A second model (model 2) was run with stabilometric measures, including mediolateral (SwayML) and anterior–posterior sway (SwayAP), velocity of antero-posterior (VelAP) and medio-lateral sway (VelML), and absolute position of center of pressure (CopX abs). A third stepwise regression model was run including all variables, resulting in a model with SwayML, BBS, and BI (model 3). Finally, correlations between independent variables were analyzed.

**Results:**

The area under the curve (AUC) for model 1 was 0.68 (95%CI: 0.53–0.83, sensitivity = 95%, specificity = 39%) with prediction accuracy of 63.3%. Model 2 resulted in an AUC of 0.68 (95%CI: 0.53–0.84, sensitivity = 76%, specificity = 57%) with prediction accuracy of 65.3%. The AUC of stepwise model 3 was 0.74 (95%CI: 0.60–0.88, sensitivity = 57%, specificity = 81%) with prediction accuracy of 67.4%. Finally, statistically significant correlations were found between clinical variables (*p* < 0.05), only velocity parameters were correlated with balance performance (*p* < 0.05).

**Conclusion:**

A model combining BBS, BI, and SwayML was best at identifying faller status in persons in the chronic phase post stroke. When balance performance is poor, a high SwayML may be part of a strategy protecting from falls.

## Introduction

People with hemiparesis following stroke have various neuromotor and sensory disorders that can lead to balance problems and falls during activities of daily living. Their risk of falling is up to triple that of an age-matched population, making fall prevention an important healthcare goal (1–4). Accurate identification of the pathological and functional factors contributing to balance disorders in persons with stroke is of utmost importance in providing adequate appropriate treatment and reducing the risk of falls (5). Postural control and balance have been extensively studied with clinical measures concerning risk of falling (3, 6–10). Simpson and colleagues followed a study population for 1 year after a stroke and found that balance was the only common independent predictor of falls in persons with stroke (11). The difference in fall rates could be explained by the difference in balance scores on clinical scales. Similarly, Mackintosh and colleagues reported reduced mobility and balance in recurrent faller’s post-stroke (9), while on the contrary Hyndman and colleagues found no differences between fallers and non-fallers using clinical scales (10).

Overall, clinical measures focusing on balance performance have proven to be only moderately good at identifying fallers or those at risk of falling. However, since balance with its underlying body functions is a complex construct it is possible that adding information from objective balance control measures might give a more complete picture of balance. This would improve our understanding of factors most likely to impact on fall risk in persons post stroke (12). Stabilometric platform measures give information on weight bearing symmetry, amount of sway, and velocity of sway during quiet standing and may give added insight into the specific underlying abnormalities in postural control and the consequential imbalance leading to falls. There are some indications that stabilometric measures related to mediolateral sway and velocity of sway, are associated with falls in healthy elderly persons, with several studies reporting an association of falls with increased mediolateral sway and increased velocity of antero-posterior sway with eyes open and closed in that population (13–18). Differences have been found in weight bearing symmetry and postural sway parameters between healthy subjects and persons with stroke, with the latter having larger and faster sway, especially in the frontal plane (10, 19–21). However, studies on the relationship of these postural impairments to the occurrence of falls in persons with stroke have reported rather ambivalent results (22). Sackley et al. found a significant relationship between increased body sway and the number of falls in persons with stroke, with however, only a small amount of the variation in the number of falls explained by body sway (23). In a study by Jørgensen instead, larger body sway did not result being a significant risk factor for falls (1). Similarly, in a recent study, Bower and colleagues found that quiet standing body sway parameters did not predict falls in the subacute phase after stroke (24). On the other hand, Lee and Jung reported postural sway at 3 months post-stroke as contributing to increased risk of falls at around 1-year post-stroke (25). As is evident, no clear relationships have been identified between postural sway impairments and falling in persons in the chronic phase after stroke and, to our knowledge, no studies have put together clinical and stabilometric measures in faller prediction models for that population (25, 26). Given the importance of improving detection of fallers and identification of potential fall risk markers, the primary aim of this study was to investigate the relative accuracy of commonly used clinical measures in stroke for identifying faller status, and the added value of quantitative measures of postural sway in quiet standing. For that purpose, we included measures of balance and mobility performance, as well as, as well as quantitative measures of postural sway in predictive models. Further, associations between clinical and stabilometric variables were explored.

## Methods

In this cross-sectional study, data from 49 persons with hemiparesis after stroke were retrospectively analyzed from a convenience sample recruited in [redacted] between January 2008 and July 2021. Participants had both clinical and stabilometric data from larger studies. The study was conducted in accordance with the Declaration of Helsinki and all participants had signed an informed consent form.

All participants met the following inclusion criteria: ≥18 years, diagnosed with first ischemic or hemorrhagic unilateral stroke, able to walk 10 m with or without an assistive device, able to provide informed consent and follow instructions. Exclusion criteria included the presence of any other neurological diagnosis or orthopedic complications that could affect balance control.

Before being assessed, all participants provided demographic information including age, stroke onset, and affected side. The history of falls (with and without complications) in the preceding 6 months was collected. The entire balance and mobility assessment procedure was carried out in one sitting by an experienced assessor, with the tests performed in random order. Participants were allowed to rest as needed during all phases of the evaluation.

### Dependent variable

Predicted variable was faller status. Participants were categorized in “fallers” (one or more falls in the prior 6 months) vs. “non-fallers” (no falls reported in the prior 6 months). A fall was defined as “an episode of unintentionally coming to rest on the ground or lower surface that was not the result of dizziness, fainting, sustaining a violent blow, loss of consciousness, or other overwhelming external factor” (27).

### Independent variables

Predictive variables were selected from commonly used clinical scales and stabilometric measures used to characterize balance performance and function of persons with stroke. In particular, included variables were related to static and dynamic balance performance, individual confidence in activities of daily living, functional independence, and frontal or sagittal plane measures of sway that have been indicated in relation with fall risk in the literature.

#### Clinical explanatory measures

Clinical variables, assessing balance and mobility, were the following: The Berg Balance Scale (BBS), the Dynamic Gait Index (DGI), the Barthel Index (BI), and the Activities Specific Balance scale (ABC).

The BBS is a 14-item test measuring balance during standing activities with a total score of 56 indicating perfect balance. The BBS has been used in many studies as a gold standard for balance scales (28). The DGI is a valid and reliable clinical measure of individual’s dynamic balance as well as his ability to walk in different conditions (e.g., walking with head turns or changing speeds) (28). Each of the eight items is scored on a scale of 0 (severe limitation) to 3 (normal performance) points, with a best score of 24 points (29, 30). The BI evaluates the functional independence with a score of best 100 meaning complete independence (31). The ABC is a 16-item scale evaluating the individuals’ confidence in not losing balance during various activities of daily living. It is composed of 16 items (ranging from 0 to 100) with a total score calculated as the mean of the items (higher scores indicate better confidence in balance performance) (32, 33).

#### Stabilometric explanatory measures

Stabilometric variables were collected on a monoaxial platform (Prokin 252, Tecnobody©) (20, 34). The platform has a circular surface of 50 cm of diameter managed by an electro-pneumatic system thorough an electronic pressure regulator. It consists of four strain gages with a sampling frequency of 20 Hz. Participants were tested wearing their normal shoes in a quiet standing position, and the position of the feet was standardized using a V-shape frame (the medial borders of the feet against the frame and the distance between the malleoli was 3 cm). Participants were asked to stand upright without any support with eyes open for 30 s (20). Instant positions of the Center of Pressure (CoP) were computed to calculate the following parameters: mean sway on the anterior–posterior (SwayAP, mm) and medio-lateral (SwayML, mm) axis; calculated as the standard deviation of raw AP and ML CoP position; velocity of sway in the anterior–posterior (VelAP, mm/s); medio-lateral (VelML, mm/s) axis, computed as the first time derivative of CoP displacements; and absolute position of CoP in mediolateral direction (CopX abs, mm; higher values indicate greater asymmetry).

### Data analysis

Descriptive statistics consisted of group medians and interquartile ranges (IQRs) of demographics and measurements. Bivariate and multivariate logistic regression analyses were carried out using faller status (0 = non-faller; 1 = faller) as the dependent variable and clinical and stabilometric explanatory measures as independent variables. Odds ratios (ORs) and related 95% confidence intervals (95%CIs) were calculated. The first multivariate model included only clinical scales as predictors (model 1); while the second multivariate model included only stabilometric variables as predictors (model 2). Backward stepwise logistic regression (model 3) was then used to identify the best predictors of fall status among all clinical and stabilometric variables (35). In model 3, variables were excluded at each step based on values of *p*, and the total number of variables was determined according to the Akaike Information Criterion (AIC). To improve stability and control for variance inflation, independent variables were removed when collinearity was of concern.

The area under the curve (AUC) in a Receiver Operating Characteristic (ROC) analysis were used to test the goodness-of-fit and accuracy of prediction of each model. An area of 1 implies optimal prediction accuracy, whereas an area ≤ 0.5 indicates that the model’s predictions are no better than would be obtained by chance. The models were evaluated at different cut-off values to determine the maximum sum of sensitivity and specificity as the optimal cut-off value (36).

Finally, Pearson’s correlations were run between the clinical and stabilometric variables included in the bivariate analyses with the value of *p*s corrected for multiple inference using Holm’s method. A significance level of *p* < 0.05 was set for all tests. All analyses were performed using R (version 4.1.0).

## Results

The characteristics of the 49 participants are described in Table 1. Thirty had a right hemiparesis and 32 were males. Twenty-one of the 49 participants (43%) were classified as fallers, while the remaining 28 (57%) were classified as non-fallers. Among the non-fallers, 21 out of 28 (75%) used an assistive device, while of the fallers 14 out of 21 (67%) used an assistive device.

**Table 1:** Demographic characteristics, Mean (SD).

	Overall	Non-fallers	Fallers	OR	95% CI	Value of *p*
	*N* = 49	*N* = 28 (57%)	*N* = 21 (43%)			
Age (years)	63.78 (12.31)	65.09 (9.42)	62.03 (15.43)	0.98	0.93–1.03	0.39
Gender						
Female	17 / 49 (35%)	9 / 28 (32%)	8 / 21 (38%)	-	-	-
Male	32 / 49 (65%)	19 / 28 (68%)	13 / 21 (62%)	0.77	0.23–2.55	0.67
Right/left side						
Left	19 / 49 (39%)	11 / 28 (39%)	8 / 21 (38%)	-	-	-
Right	30 / 49 (61%)	17 / 28 (61%)	13 / 21 (62%)	0.95	0.29–3.04	0.93
Assistive device						
No	14/49 (29%)	7/28 (25%)	7/21 (33%)	-	-	-
Yes	35 / 49 (71%)	21/28 (75%)	14/21 (67%)	0.54	0.15–1.85	0.33
Disease duration (years)	2.68 (5.29)	1.86 (1.85)	3.69 (7.62)	1.10	0.96–1.42	0.33
BBS (score)	44.10 (7.30)	46.00 (7.35)	41.57 (6.58)	0.91	0.83–0.99	0.04
DGI (score)	13.27 (5.34)	14.29 (5.52)	11.90 (4.88)	0.91	0.81–1.02	0.13
BI (score)	92.29 (11.05)	95.04 (6.29)	88.62 (14.66)	0.94	0.87–1.00	0.07
ABC (score)	58.72 (22.08)	61.71 (22.22)	54.79 (21.79)	0.99	0.96–1.01	0.27
SwayAP (mm)	5.82 (1.57)	6.00 (1.90)	5.58 (0.97)	0.83	0.54–1.21	0.35
SwayML (mm)	4.12 (2.12)	4.44 (2.26)	3.68 (1.89)	0.83	0.60–1.10	0.22
VelAP (mm/s)	17.12 (5.54)	16.35 (5.23)	18.14 (5.89)	1.06	0.96–1.20	0.27
VelML (mm/s)	8.70 (3.59)	8.65 (3.91)	8.76 (3.21)	1.01	0.86–1.18	0.92
CopX abs (mm)	21.24 (16.13)	23.33 (16.06)	18.46 (16.20)	0.98	0.94–1.02	0.30

OR, odds ratio; BBS, berg balance score; DGI, dynamic gait index; BI, barthel index; ABC, activities-specific balance confidence; SwayAP, amplitude of sway in anteroposterior direction; SwayML, amplitude of sway in mediolateral direction; VelAP, velocity of sway in anteroposterior direction; VelML, velocity of sway in mediolateral direction; and CopX abs: absolute position of center of pressure in mediolateral direction.

### Bivariate analysis

Table 1 also shows OR and 95%CI from bivariate logistic regression analyses for independent variables. The only significant OR of clinical variables was found for the BBS (OR = 0.91, *p* = 0.04), while both OR for the BI (OR = 0.94, *p* = 0.07) and the DGI (OR = 0.91, *p* = 0.13) were non-significant. The OR of stabilometric variables were non-significant for the SwayML (OR = 0.83, *p* = 0.22), with fallers having smaller SwayML than non-fallers, and VelAP (OR = 1.06, *p* = 0.27), with fallers having higher velocity of sway.

### Multivariate analysis

Table 2 shows the multivariate logistic regressions of clinical (model 1) and stabilometric (model 2) variables. Table 2 also shows the backward stepwise linear regression model including both clinical and stabilometric variables (model 3). In models 2 and 3, the independent variable VelML was excluded from the regression analysis due to collinearity.

**Table 2:** Prediction models of faller/non-faller using clinical (model 1), stabilometric (model 2), and clinical and stabilometric variables selected by backward stepwise regression analyses (model 3).

Model 1: Clinical variables				
	β	OR	95%CI	*p* value
Independent variables				
(Intercept)	8.79			
BBS	−0.12	0.89	0.75–1.03	0.14
BI	−0.06	0.95	0.86–1.02	0.19
DGI	0.09	1.10	0.87–1.40	0.43
ABC	0.003	1.00	0.97–1.04	0.87
**AIC: 70.00; accuracy: 63.3%**				
**Model 2: Stabilometric variables**				
	β	OR	95%CI	*p* value
Independent variables				
(Intercept)	−0.12			
SwayAP	−0.28	0.76	0.42–1.29	0.32
SwayML	−0.13	0.88	0.49–1.55	0.66
VelAP	0.13	1.14	0.99–1.36	0.10
XcopABS	−0.01	0.98	0.92–1.05	0.63
**AIC: 71.30; accuracy: 65.3%**				
**Model 3: Stepwise regression**				
	β	OR	95%CI	*p* value
Independent variables				
(Intercept)	10.35			
BI	−0.05	0.95	0.87–1.02	0.18
BBS	−0.09	0.91	0.81–1.01	0.09
SwayML	−0.38	0.68	0.44–0.97	0.06
**AIC: 64.26; accuracy: 67.4%**				

OR, odds ratio; BBS, berg balance scale; BI, barthel Index; DGI, dynamic gait index; ABC, activities-specific balance confidence; AIC, akaike information criterion; SwayAP, amplitude of sway in anteroposterior direction; SwayML, amplitude of sway in mediolateral direction; VelAP, velocity of sway in anteroposterior direction; CopX abs, absolute position of center of pressure in mediolateral direction; VelML, velocity of sway in mediolateral direction.

In clinical model 1, the BBS and the BI were the strongest predictors of faller status with respective OR = 0.89 (95%CI: 0.75–1.03) and OR = 0.95 (95%CI: 0.86–1.02), while in the stabilometric model 2, the only nearly significant independent predictor was VelAP with an OR = 1.14 (95%CI: 0.99–1.36).

Finally, starting from all the clinical and stabilometric variables included in the bivariate analyses, a backward stepwise logistic regression model was performed. The model with predictors selected by stepwise regression (model 3) included SwayML, the BBS, and the BI. SwayML showed a moderate inverse relation with the faller status (OR = 0.68, 95%CI: 0.44–0.97), while both the BBS and the BI showed weaker relations with the faller status (BBS: OR = 0.91, 95%CI: 0.81–1.01; BI: OR = 0.95, 95%CI: 0.87–1.02).

**Figure 1:**
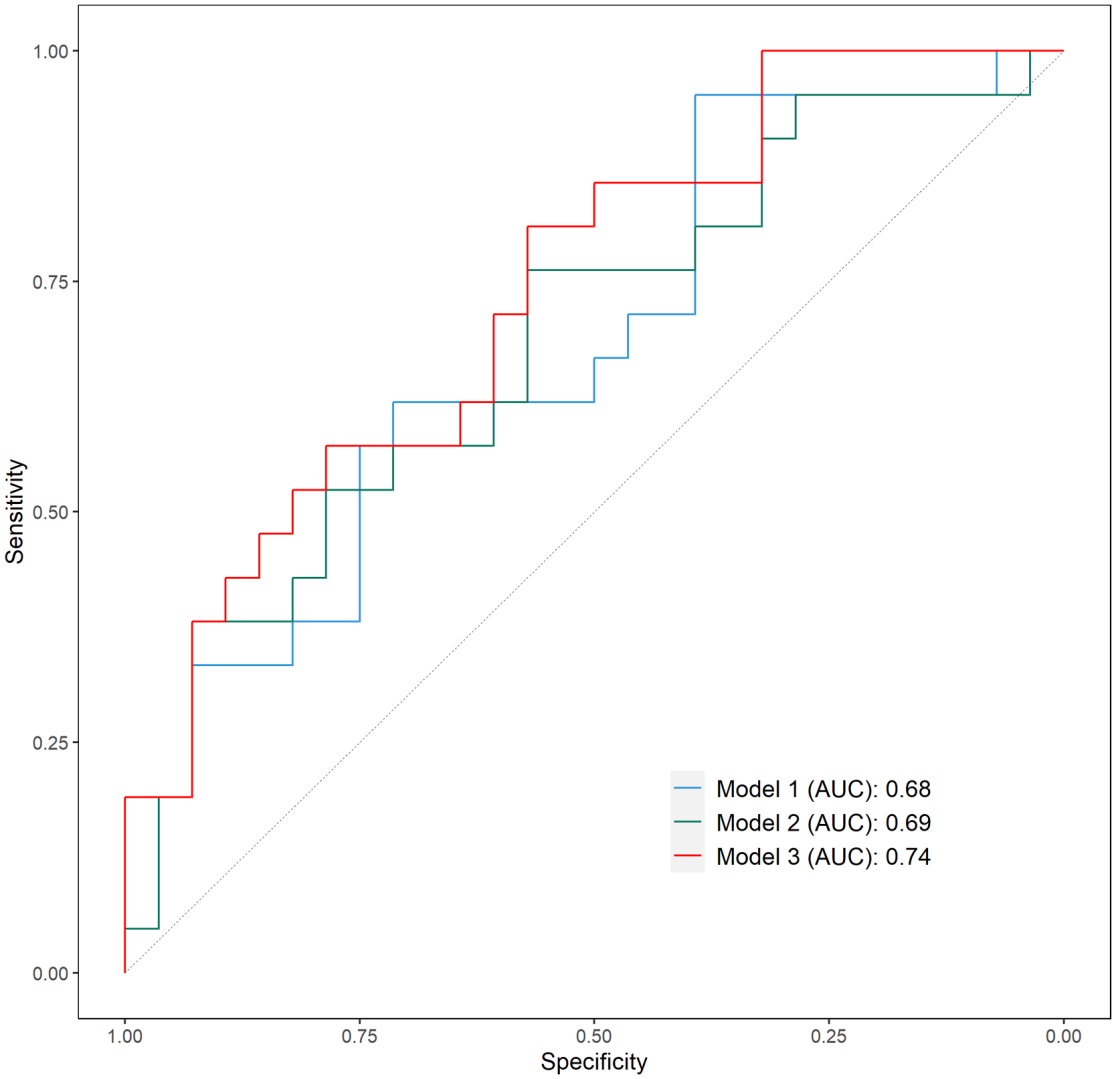
Receiver operating characteristic (ROC) curves of model 1, model 2, and model 3. Sensitivity: percentage of fallers correctly identified; Specificity: percentage of non-fallers correctly identified.

The AUC of model 1 (Figure 1) tested in the ROC analysis, reflecting the model’s ability to correctly classify faller status, was 0.68 (95%CI: 0.53–0.83) with sensitivity and specificity of 95 and 39%, respectively; the AIC, reflecting the model’s prediction error, was equal to 70.0; and accuracy of prediction was 63.3%. The AUC for model 2 instead was 0.68 (95%CI: 0.53–0.84) with sensitivity and specificity of 76 and 57%, respectively; the AIC was equal to 71.3, and prediction accuracy was 65.3%. Finally, the AUC for model 3 was 0.74 (95%CI: 0.60–0.88) with maximum sensitivity and specificity of 57 and 81%, respectively; the AIC was equal to 64.26, and prediction accuracy was 67.4%.

### Correlation analysis

Table 3 shows Pearson’s correlations between variables. All clinical measures were significantly correlated (*p* < 0.05) among them, with the highest correlation between the BBS and the DGI (*r* = 0.84, *p* < 0.001). SwayML correlated highly with VelML (*r* = 0.67, *p* < 0.001) and with CopX abs (*r* = 0.73, *p* < 0.001) that were also highly correlated among them (*r* = 0.79, *p* < 0.001). The VelML and VelAP were correlated (*r* = 0.67, *p* < 0.001). The BBS and the DGI correlated moderately with VelML (*r* = −0.51, *p* < 0.05; *r* = −0.63, *p* < 0.001, respectively) and the BBS had a low correlation with VelAP (*r* = −0.47, *p* < 0.05).

**Table 3:** Pearson’s correlations between clinical and stabilometric variables.

DGI	0.84^**^							
BI	0.50^*^	0.56^*^						
ABC	0.54^*^	0.61^**^	0.41					
SwayAP	−0.13	−0.19	−0.11	−0.07				
SwayML	−0.20	−0.33	−0.16	−0.16	0.57^*^			
VelAP	−0.47^*^	−0.40	−0.13	−0.26	0.31	0.22		
VelML	−0.51^*^	−0.63^**^	−0.25	−0.31	0.44^*^	0.70^**^	0.67^**^	
CopX	−0.34	−0.41	−0.16	−0.14	0.27	0.73^**^	0.28	0.79^**^
	BBS	DGI	BI	ABC	SwayAP	SwayML	VelAP	VelML

^**^p < 0.001; ^*^p < 0.05; BBS, berg balance scale; BI, barthel Index; DGI, dynamic gait index; ABC, activities-specific balance confidence; SwayAP, amplitude of sway in anteroposterior direction; SwayML, amplitude of sway in mediolateral direction; VelAP, velocity of sway in anteroposterior direction; and CopX abs: absolute position of center of pressure in mediolateral direction.

**Figure 2:**
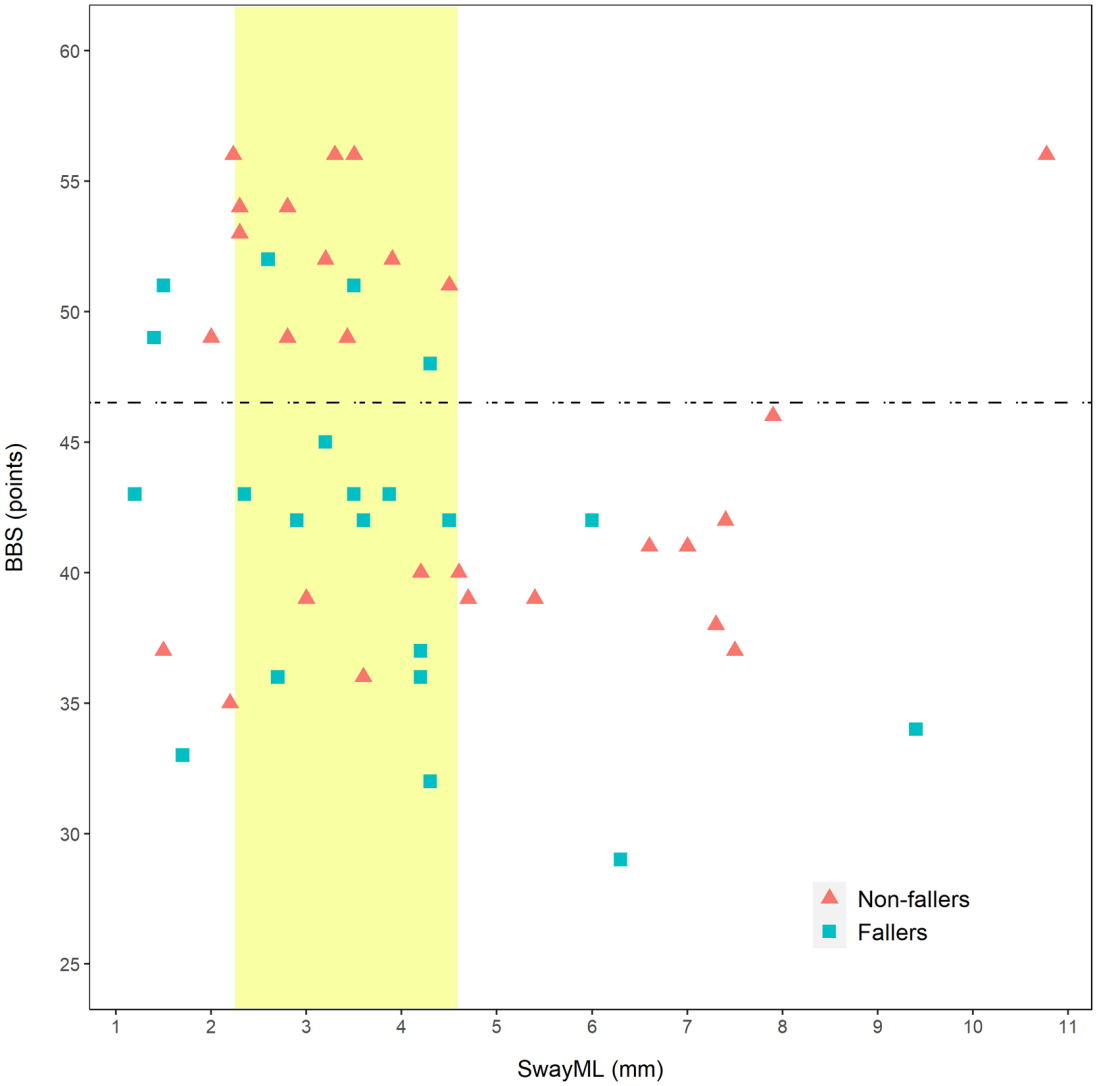
Scatterplot of relation of Berg Balance Scale and SwayML with faller status. BBS, Berg Balance Scale; SwayML, amplitude of sway in mediolateral direction; horizontal dashed line, fall risk cut-off value of BBS (46.5 points); and vertical band, SwayML range of values corresponding to normative data (2.25–4.59 mm).

Finally, the relation between SwayML, BBS, and fallers status was further investigated graphically (Figure 2). The fall risk cutoff value of BBS (46.5 points) and normative data of SwayML (2.25–4.59 mm) were added to the graphic to better characterize the participants performance (37, 38).

## Discussion

This cross-sectional study yielded a risk model for the identification of faller status of persons in the more chronic post-stroke state. A model including both stabilometric and clinical parameters, the SwayML, the BBS and the BI, resulted better in correctly identifying retrospective fallers among people in the chronic phase after stroke than did models that included only clinical or stabilometric parameters, respectively.

The percentage of fallers in this study was 43%, which is similar or somewhat lower than that reported in other retrospective studies of fall history and balance in persons post-stroke (3, 11, 20, 39, 40), and similar to a prospective study conducted for 3 months after discharge from inpatient rehabilitation (41). Regarding clinical measures, fallers had significantly lower total scores on BBS compared with non-fallers, while the BI, DGI, and ABC did not differ between faller groups. The first model containing these clinical measures (model 1) resulted in a prediction accuracy of approximately 63%, indicating that it was only moderately effective in identifying faller status. Specifically, it was very good at identifying a faller with a high sensitivity (95%), while it was not at all good at identifying a non-faller (37%).

In the literature, lower BBS scores have been associated with a higher probability of falling in persons with stroke, for example, Simpson and colleagues found lower BBS to be predictive of falls at 1 year post-stroke (11, 40, 42–44). On the contrary, Harris and colleagues found no association between falls history and BBS scores in persons with chronic stroke (7). Together with our findings, this suggests that while reduced balance performance may influence the risk of falling other factors contribute to their falling. These may include functional independence in daily life activities, fear of falling, as well as, aspects of postural control (45–47).

Regarding the stabilometric measures, there were no significant differences between faller groups, although there was a tendency for higher values in SwayML in non-fallers, with over 20% more sway distance than fallers. Fallers instead had SwayML values closer to those of healthy subjects reported elsewhere (37). Similarly, the absolute stance positioning was more asymmetric in the non-faller group by about 26%. The model containing only the stabilometric variables (model 2) resulted in a moderate prediction accuracy of approximately 65%, indicating that it was slightly better at identifying faller status than a model containing often used clinical scales of balance and functional independence. In addition, model 2 was more balanced, with a good sensitivity for faller identification (76%) and with a better specificity than model 1 (57%). The accuracy was similar to model 1 (67.4%).

The stepwise regression model combining all clinical and stabilometric measures resulted in SwayML, BBS, and BI in the final model (model 3). Model 3 presented the best discriminative ability of faller status (accuracy =74%), with BBS and SwayML being the strongest predictors in the model. Model 3 was slightly more accurate than the other two models, indicating an advantage of combining clinical and stabilometric parameters in faller identification.

SwayML was the most significant predictor in model 3 and had an inverse relation with being a faller. This could indicate an increased probability of being a faller with a near normal physiological amplitude of sway in the frontal plane, which contrasts with another study on neurologically healthy elder population that reported an increased risk of falls with high amplitudes of SwayML (13). Meanwhile, other studies on the stroke population did not identify SwayML as a risk factor for falls (1, 6, 22). Our results are interesting considering findings of Marigold and Eng of greater asymmetry in quiet standing being related to increased SwayML in persons with stroke (48). Their study also found that persons with milder asymmetry had greater visual dependence than those with more asymmetry and increased SwayML. It is possible that closer to normal SwayML is found in persons post-stroke that put a more symmetric or greater load on the more affected limb, with the limb often acting as a fixed strut with excessive co-contraction around the knee; and that these persons potentially rely excessively on visual information to correct their asymmetry (7, 49). Regarding the increased SwayML we found in our persons post-stroke that did not fall, as suggested by Park and colleagues, some persons post-stroke might implicitly prefer a natural asymmetry in standing and walking in order to not compromise balance (50).

When we looked more closely at the relation between the parameters that discriminated between fallers and non-fallers in the bivariate analysis, SwayML and BBS (see Figure 2), it was evident that persons with a score higher than 46 on the BBS tended to have a near normal SwayML. On the other hand, we saw a tendency for non-fallers with a low BBS to have higher SwayML values and interestingly there were several fallers that had relatively normal sway values. It could mean that in some persons with poorer balance, an increase in SwayML may be a protective measure, and that it likely comes with an increase in asymmetry toward the better leg. Since symmetry in standing and walking is often a rehabilitation goal in persons post-stroke the above are important concepts for further studies.

Regarding the associations between the variables of the bivariate models, we found moderate to strong linear correlations among the clinical variables. A higher BBS was associated with a low VelAP in accordance with findings from the literature (13, 49). SwayML, which was the best single indicator of faller status in model 3, had no relation with either BBS, BI, or VelAP confirming findings of others (49, 51).

Asymmetry in standing, as an absolute value in the mediolateral direction (CopX abs) toward the less affected leg, did not discriminate between fallers and non-fallers in bivariate analyses, but it did correlate highly and positively with SwayML indicating a relationship between the two parameters as suggested by previous findings in the literature (48).

Asymmetric stance may be an unconscious strategy to protect against fall risk. By keeping the CoP closer to the healthy leg, the non-fallers are more protected against unexpected events that might otherwise lead to falls. At this point, we urgently need prospective studies to further verify the predictive value of SwayML during quiet standing combined with clinical variables in predicting a future fall risk. Based on our results, it would be most interesting to study for persons post stroke at a higher risk of fall. Such a prediction model could have a great importance for fall prevention programs and should be further investigated.

Our study adds to the literature as it is the first study to put together clinical variables and stabilometric measures to predict faller status in persons with stroke in chronic phases. However, the study has some limitations. Fall incidence was retrospective and self-reported and although this methodology is commonly used in cross-sectional studies, the effect of this reporting mechanism is questionable. Efforts were made to minimize this bias by verifying with caregivers (including partners, colfs, sons, and daughters) the answers given by participants. Further, since the fall reports were retrospective, the predictive value of the measures cannot be assumed.

To further validate the findings of this study and its resultant implications for identification of fall risk factors, prospective designs with appropriate methods of analysis, and intervention studies addressing factors influencing amount of sway in the ML direction are needed. These should consider also different disability and chronicity levels.

An important limitation of the present study is the inclusion of only quiet standing postural sway measures. Reactive balance measures in response to self-induced and external balance perturbations and mobility related balance measures should be included in future studies looking to identify risk factors related to falls in persons post-stroke. The use of stabilometric platforms in general for postural assessment of persons with stroke might not always be economically feasible. However, given that they seem to add value to the description of balance and faller identification their use should be recommended when possible.

In conclusion, greater understanding of the relative contribution of risk factors to falls after stroke can lead to the development of better fall prevention programs. While clinical measures of balance or stabilometric measures alone were reasonably good at predicting faller status in a logistic regression, a model combining clinical and stabilometric measures had a better accuracy of faller status. Counterintuitively, more physiological amplitudes of SwayML during quiet standing resulted in the strongest fall predictor in the final model. Further investigation showed that this was more likely in persons with higher balance disability. This information is important since it is the first time this relation is identified in people with stroke that are fallers. It also has important implications for future directions in balance rehabilitation for persons with stroke. Focusing on symmetry in standing in persons with poor balance performance may not be the best approach if fall prevention is of importance.

## Data availability statement

The raw data supporting the conclusions of this article will be made available by the authors, without undue reservation.

## Ethics statement

Ethical review and approval was not required for the study on human participants in accordance with the local legislation and institutional requirements. The patients/participants provided their written informed consent to participate in this study.

## Author contributions

JJ and DC designed, planned, and supervised the study. FM and CC performed clinical and stabilometric assessments. AT and DC performed all statistical analyses. JJ drafted the entire manuscript with input of all the co-authors. All authors contributed to the article and approved the submitted version.

## Funding

This study was supported and funded by the Italian Ministry of Health—Ricerca Corrente 2022.

## Conflict of interest

The authors declare that the research was conducted in the absence of any commercial or financial relationships that could be construed as a potential conflict of interest.

## Publisher’s note

All claims expressed in this article are solely those of the authors and do not necessarily represent those of their affiliated organizations, or those of the publisher, the editors and the reviewers. Any product that may be evaluated in this article, or claim that may be made by its manufacturer, is not guaranteed or endorsed by the publisher.
